# Autonomic Nervous System Adaptation and Circadian Rhythm Disturbances of the Cardiovascular System in a Ground-Based Murine Model of Spaceflight

**DOI:** 10.3390/life13030844

**Published:** 2023-03-21

**Authors:** Ophélie Hélissen, Marc Kermorgant, Sébastien Déjean, Aurélie Mercadie, Sophie Le Gonidec, Rana Zahreddine, Denis Calise, Nathalie Nasr, Céline Galès, Dina N. Arvanitis, Anne Pavy-Le Traon

**Affiliations:** 1 Institute of Cardiovascular and Metabolic Diseases, UMR1297, INSERM, University Hospital of Toulouse, 31400 Toulouse, France; ophelie.helissen@gmail.com (O.H.);; 2 Neurology Department, University Hospital of Toulouse, 31400 Toulouse, France; 3 Institut de Mathématiques de Toulouse, UMR5219, CNRS, Université de Toulouse, UT3, 31062 Toulouse, France; 4 CREFRE-Anexplo, Services Phénotypage et Microchirurgie, UMS006, INSERM, Université de Toulouse, UT3, ENVT, 31062 Toulouse, France

**Keywords:** microgravity, hindlimb unloading, telemetry, autonomic nervous system, circadian rhythm

## Abstract

Whether in real or simulated microgravity, Humans or animals, the kinetics of cardiovascular adaptation and its regulation by the autonomic nervous system (ANS) remain controversial. In this study, we used hindlimb unloading (HU) in 10 conscious mice. Blood pressure (BP), heart rate (HR), temperature, and locomotor activity were continuously monitored with radio-telemetry, during 3 days of control, 5 days of HU, and 2 days of recovery. Six additional mice were used to assess core temperature. ANS activity was indirectly determined by analyzing both heart rate variability (HRV) and baroreflex sensitivity (BRS). Our study showed that HU induced an initial bradycardia, accompanied by an increase in vagal activity markers of HRV and BRS, together with a decrease in water intake, indicating the early adaptation to fluid redistribution. During HU, BRS was reduced; temperature and BP circadian rhythms were altered, showing a loss in day/night differences, a decrease in cycle amplitude, a drop in core body temperature, and an increase in day BP suggestive of a rise in sympathetic activity. Reloading induced resting tachycardia and a decrease in BP, vagal activity, and BRS. In addition to cardiovascular deconditioning, HU induces disruption in day/night rhythmicity of locomotor activity, temperature, and BP.

## 1. Introduction

The ability of Humans to explore space is limited by both psychological factors such as isolation and confinement as well as physical factors such as exposure to radiation and microgravity [1,2]. Numerous studies aiming to understand the biological impact of spaceflight focused largely on the risks of microgravity, which has important physiological consequences on the human body. Astronaut physiology is challenged in three phases: first, changes elicited upon entry to microgravity (short-term adaptation); second, the effects of prolonged exposure to microgravity; third, the re-adaptation to Earth’s gravity upon return. A number of studies from exposure to microgravity have shown that immediate loss of orthostatic pressure gradients leads to fluid redistribution towards the upper part of the body [3,4]. On return to Earth, the astronauts showed neuro-vestibular disturbances, reduction in bone mass [5,6], deconditioning of the musculoskeletal system [7], dysregulation of the immune system [8], sleep problems with circadian misalignment [9] and cardiovascular deconditioning [10]. Few studies have been performed to assess the autonomic nervous system (ANS) inflight, which plays a key role in cardiovascular adaptation to postural challenge [11]. Early short flight studies showed reduced baroreflex sensitivity (BRS) and evidence of increased sympathetic activity [12]. However, the role of the ANS during adaptation to short and long duration missions remains unclear [12]. Blunted sympathetic responses and compromised regulation of the ANS, persisting upon returning to Earth, could be factors of orthostatic intolerance and resting tachycardia [4,13,14]. On board, real microgravity-based studies are mainly limited due to high costs and difficulties in the collection of data during missions.

Despite their limitations, ground-based analogs of spaceflight have proven to be valuable techniques regarding numerous physiological systems and correlate with findings observed from real microgravity experimentation [15,16,17,18,19]. Rodent studies for microgravity often use the model referred to as hindlimb unloading (HU), which has proven to bring about similar physiological adaptations as observed in astronauts from real microgravity settings [20,21]. Some of these physiological alterations include fluid redistribution to the upper part of the body, increased natriuresis/diuresis leading to hypovolemia and hemodynamic dysregulation with resting tachycardia post-HU [20,21,22]. Attenuation of the baroreflex-mediated sympathoexcitation in response to a hypotensive stimulus has been shown in both rats [23,24] and in mice [13] following HU. However, the hemodynamic and autonomic responses during simulated microgravity in rodent models are controversial due to the discrepancy between species but also because of differences in both the duration of microgravity exposure as well as the choice of time points when measurements are recorded.

The purpose of this study is to clarify various controversial aspects of these forms of experiments by devising a murine HU model with continuous monitoring of relevant parameters. We aimed to define the kinetics of cardiovascular adaptation and recovery using a mouse model with three phases over 10 days: 3 days of control, 5 days of HU and 2 days of recovery. Over the time course, we measured mouse weight as well as their water and food intake. Using implantable radio telemetry devices, we continuously collected data on mouse subcutaneous temperature and locomotor activity, as well as cardiac parameters, arterial blood pressure (ABP), and HR. The cardiac parameters were further exploited to calculate heart rate variability (HRV) and BRS.

Our study showed that HU induced: (1) an initial bradycardia associated with an increase in vagal indirect markers of ANS activity, followed by a return to baseline values; (2) a persistent striking decrease in locomotor activity and in mice temperature associated with a disruption of circadian rhythms; (3) an increase in BP associated to a disruption of circadian rhythms suggestive of an increase in sympathetic activity; (4) a resting tachycardia at the recovery with a decrease in BP and in vagal activity markers reflecting the cardiovascular deconditioning.

## 2. Materials and Methods

### 2.1. Ethics Statement

Animal procedures were in accordance with European Communities Council Directive (2010/63/EU) and European Community standards on the Care and Use of Laboratory Animals (Ministère de l’Enseignement supérieur, de la Recherche et de l’Innovation, MESRI, France, authorization 25468).

### 2.2. Animals

C57BL/6J mice (12-week-old males) were purchased from Charles River Laboratories (Saint Germain l’Arbresle, France). Prior to experimentation, the mice were housed for 1 month in standard ventilated cages in the animal facility (US006 CREFRE Rangueil, Toulouse, France) under controlled conditions, with 23 ± 1 °C of ambient temperature and 60% of relative humidity. They were synchronized to a 12-h light-dark cycle (light cycle (LC) from 7:00 a.m. to 7:00 p.m., dark cycle (DC) from 7:00 p.m. to 7:00 a.m.) with access to standard mouse chow (R04-10, SAFE^®^, Rosenberg, Germany) and water ad libitum. One week prior to experimentation, mice were separated and individually housed in the cages used for HU. Mice (*n* = 16) were divided into two-independent groups: 10 mice were used for telemetry recordings for ABP, HR, locomotor activity and subcutaneous temperature in two independent periods (*n* = 5 each) and 6 mice were used for central (core) temperature recording.

### 2.3. Surgery

Mice were implanted with the HD-X11 transmitters (Data Science International^®^, DSI, Saint Paul, MN, USA). Briefly, mice were anaesthetized with ketamine (100 mg/kg) and xylazine (10 mg/kg) by intraperitoneal injection (with 26 G needle) followed by a subcutaneous injection of buprenorphine (100 μg/kg). Anesthesia was maintained by mask inhalation of vaporized isoflurane (1.5%) and 100% oxygen mixed (0.2 L/min). Mice were placed on a warmed bed and the depth of anesthesia was assessed regularly by hind leg pinch. A midline incision (1.5 cm) was made on the ventral neck. The left common carotid artery was isolated and ligated to its bifurcation. A second ligature was used to clamp on the artery 6 mm proximal to the site of ligation. By a small incision in the artery wall, the catheter was advanced 7–10 mm towards the aorta and fixed in place with silk sutures (6-0 surgical suture, Ethicon, Somerville, NJ, USA). A subcutaneous pocket on the left flank of the animal was made with blunt scissors, and the body implant was inserted. Two small cutaneous incisions (3 mm) in the skin were performed on both sides of the heart, and the two electrodes of the telemetry transmitter from the ECG recording are tunneled under the skin. ECG electrodes were placed and sutured subcutaneously with 6-0 surgical suture (Ethicon, Somerville, NJ, USA) with the negative electrode attached to the right pectoral muscle; the positive lead on the left side on the level of xiphoid space and caudal to the heart. Skin incisions were sutured (5-0 surgical non-absorbable suture, Ethicon, Somerville, NJ, USA) and animals were allowed to recover on a warming pad (37 °C). For the recording of core temperature, the implant was inserted into the peritoneal cavity of 6 mice. The peritoneum was opened with fine scissors, the implant body was placed, and the peritoneum was sutured with a 6-0 surgical suture. Following recovery, mice were housed in individual cages and placed on the telemetric receivers (RPC-1 PhysioTelTM Receivers, DSI, Saint Paul, MN, USA), in a light-dark cycled room for the 7-day recovery period.

### 2.4. Hindlimb Unloading

Mice were subject to −30° tail unloading as described [25]. The apparatus and cages were employed as previously described [26]. Mice were briefly anaesthetized with inhaled mixed of isoflurane (2%) and 100% oxygen gas (0.2 L/min), in order to wrap the tail to the metal rod using the adhesive tape, ensuring that there is sufficient uncovered space around the tail to ensure adequate thermoregulatory capacity. The metal rod was connected to the unloading device, composed of a pulley system and a swivel, located at the top of the cage on two parallel metal bars of the cage. Using this setup and a grid at the bottom of the cage, mice were able to access all areas of the cage and were able to eat, groom, and move easily with their forelimbs.

### 2.5. Experimental Design

**Figure 1:**
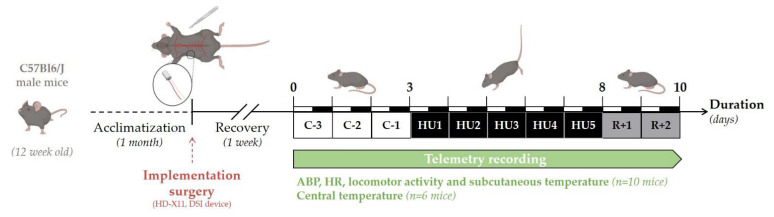
Schematic representation of the experimental design.

The experimental design of the study is depicted in Figure 1. Telemetry recordings were initiated 7 days after surgery. Baseline collection of telemetric data was performed during 3 days (Control, C-3 to C-1). All animals (*n* = 16) were suspended by their tail for a total of 5 days (Unloading, HU1 to HU5), after which they were returned to the normal four-extremity weight bearing “reloaded” position for 2 days (Recovery, R+1, and R+2). For consistency, and to minimize disturbances in the collection of the physiological data, all procedures including animal handling, cleaning, changes and measurements of food and water intake, and, data collection of recordings were performed at 10 a.m. Food (15 g per day) and water were weighed daily from surgery to the end of telemetry recordings. Body weight was monitored daily during the control period, then only at the unloading and recovery period. Body weight, food, and water intake were analyzed and described in this study for 11 mice. As previously mentioned, 16 mice were used in three independent experiments: two independent groups of 5 were used for the recording of ABP, HR, activity, and subcutaneous temperature, and 6 for the recording of core temperature. All animals survived implantation and to experimental endpoints. Technical issues included the intermittent dropout of the ECG signal on 2 recordings out of 10 mice. These mice were removed from all analyses of telemetric data. No gross pathological signs were noticed in any of the 16 mice. At the end of the protocol, telemetric devices were removed and the mice were sacrificed by cervical dislocation under anesthesia.

### 2.6. Telemetric Data Collection and Analysis

Telemetry transmitters were activated by positioning the magnet in close proximity to the implant as per the manufacturer’s instructions (Data Science International^®^, DSI, Saint Paul, MN, USA). ABP, HR, locomotor activity, and temperature (subcutaneous or intraperitoneal) signals were collected using the data acquisition system (Ponemah Physiology Platform Software, v6.41, DSI, Saint Paul, MN, USA). Blood pressure data are a direct analogue recording of the absolute intra-arterial pressure that is adjusted for variations in atmospheric pressure. HR was directly obtained from ECG and was also calculated from the blood pressure pulse. Activity counts were obtained by monitoring changes in the signal strength of the transmitter signal reflecting gross movements. This method only detects the movement of the transmitter and does not register grooming or other behavioral activities [27].

Signals were digitized at a rate of 1000 Hz. In order to remove measurement bias due to the entry of the experimenter at 10 a.m., this hour was excluded from the analysis. LC and DC values were determined with a 12-h mean.

### 2.7. Heart Rate Variability Analysis

HRV analysis was performed with the Ponemah software module (Heart Rate Variability, Ponemah, version 6.41, DSI, Saint Paul, MN, USA). HRV analysis was assessed in both time and frequency domains. Each R peak was detected and R-R intervals were determined from ECG recordings. Ectopic and artefact beats were removed. Since the signal is non-stationary in awake mice, we averaged 3 independent 3 min periods for HRV analysis as reported [28]. Therefore, each beat of three stable ECG 3 min intervals, with no erratic fluctuations, were analyzed and averaged between 8 and 10 a.m. We excluded one mouse from the HRV analyses because stable discernable 3 min intervals were not distinguished by the experimenter.

Time-domain analysis included the following parameters: the mean of R-R intervals, the standard deviation of all normal R-R intervals (SDNN), and the root-mean square differences of successive R-R intervals (RMSSD). Frequency-domain analysis was performed using Fast Fourier Transform spectrum with 1.024 spectral points series with 50% overlap using Welch’s periodogram. Segment length with linear interpolation and resampling to a 50 Hz interbeat time series and 20 ms tolerance (together with Hanning windowing) were averaged from the data recorded. Two separate spectral components were analyzed: the low frequency (LF, 0.10–1.00 Hz) and the high frequency (HF, 1.00–5.00 Hz) powers. LF and HF were expressed in absolute values of power (ms^2^). Very low frequency (VLF, 0.01–0.10 Hz) was excluded from the analysis.

### 2.8. Baroreflex Sensitivity Analysis

The BRS was calculated by the sequence technique (eCar software, Angers, France). Stable signals in 3 min intervals were analyzed (*n* = 7 mice). Consecutive increases or decreases in SBP associated with parallel changes in R-R intervals lengthening or shortenings, indicated spontaneous baroreflex responses. Up and down sequences were defined to a ramp of 3 SBP beats, associated with subsequent R-R intervals. An average number of 20 sequences (40 segments) were used for the analysis. No thresholds in SBP, R-R interval, or coefficient correlation (r) were used and the spontaneous BRS was calculated as the slope (ms/mmHg) of the linear regression lines.

### 2.9. Statistical Analysis

Changes in food consumption, water intake, body weight, 24 h mean variations, HRV, and BRS analysis, were analyzed by one-way repeated-measures of variance analyses (ANOVA, comparison to the last day of control, C-1) with Dunnett post-test comparison.

Two-way ANOVA (with Condition and Cycle factors) with Tukey post-test comparisons, were used to evaluate the interaction effect between unloading and LC/DC variations. All results were expressed as mean ± SD, and were statistically significant when *p*-values < 0.05.

Figures and statistical analyses were performed with GraphPad Prism 9 software (version 9.3.1, Boston, MA, USA) and R software (version 4.0.3, R Core Team 2022, Austria). Our continuously collected data were first averaged into 2 h time bins and plotted using R, by applying the ggplot2 package [29] (version 3.3.6), which allows for graphical displays. To better visualize trends and patterns in our times series, LOcally Estimated Scatterplot Smoothing (LOESS; [30]) was used. To gauge the extent of the change in amplitude and acrophase on oscillatory data, Cosinor based analyses were applied, and one-way ANOVA or Friedman test with Tukey or Dunn’s (respectively) post-test comparison was performed [31,32,33].

## 3. Results

### 3.1. Physiological Phenotyping

During the control period (C-3 to C-1) daily average food intake was 5.7 ± 0.9 g and water intake was 4.1 ± 1.2 g. Since there was no significant difference between the control days, we chose C-1 values as our baseline values for all comparison studies.

**Figure 2:**
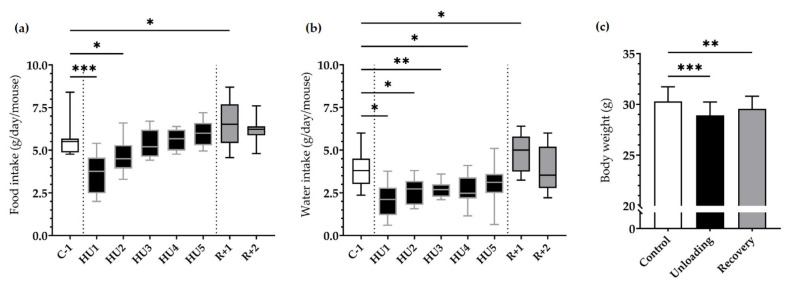
Effects of HU on food, water intake and body weight. (**a**) Food and (**b**) water intake was averaged daily during basal state (C-1, white box), during HU (HU1 to HU5, black box), and during recovery (R+1 and R+2, grey box). Data were measured 24 h, at 10 a.m. the next day (*n* = 11 mice). * *p* < 0.05, ** *p* < 0.01 and *** *p* < 0.001 vs. C-1. (**c**) Body weight was measured before unloading (control, white bar), at the reloading (unloading, black bar) and after 2 days of recovery (recovery, grey bar). Body weight values were expressed as mean ± SD (*n* = 11 mice). ** *p* < 0.01 and *** *p* < 0.001 vs. control.

HU resulted in a marked decrease in food intake at HU1 (*** *p <* 0.001), which persisted to HU2 (* *p <* 0.05) compared to C-1. This dramatic decrease was then followed by a gradual recuperation and food intake was finally similar to C-1 by the end of the unloading period. Food intake was increased at R+1 as compared to C-1 (* *p <* 0.05) (Figure 2a).

A similar pattern was observed for water intake, whereby a significant decrease at HU1 (* *p* < 0.05), HU2 (* *p* < 0.05), HU3 (** *p* < 0.01) up to HU4 (* *p* < 0.05) compared to the control C-1. Water consumption increased at R+1 (* *p* < 0.05) compared to C-1 (Figure 2b).

Body weight was decreased by 4.5% after Unloading (*** *p* < 0.001) compared to Control. Mice began to recover their body weight in the 2-day recovery period, even if by the end of our analyses they still presented a 2.4% decrease (** *p* < 0.01) compared to their initial weight (Figure 2c).

ABP, HR, activity, and temperature were directly and continuously recorded for the 10 days of the experiment. To globally assess the effects by day, we binned the data into 24 h averages. For all our analyses, we compared values to C-1 as there were no significant differences between the different control days.

**Figure 3:**
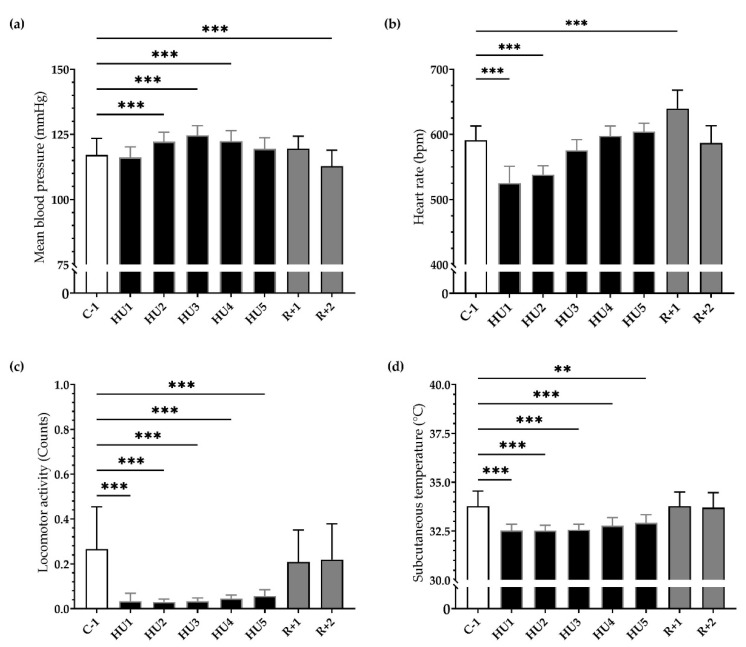
Ten-day continuous recordings of MBP, HR, locomotor activity, and subcutaneous temperature. Histograms representing day averaged data for each measure factor illustrating the values of (**a**) mean blood pressure, (**b**) heart rate, (**c**) locomotor activity, and (**d**) subcutaneous temperature. All data are expressed as 24 h mean ± SD (*n* = 8 mice). ** *p <* 0.01; *** *p <* 0.001 vs. C-1.

Our results showed a significant and progressive increase in mean ABP during the first 48 h of HU (*** *p* < 0.001) persisting to HU3 (*** *p* < 0.001) until HU4 (*** *p* < 0.001) compared to C-1. After 2 days of recovery, ABP significantly decreased compared to C-1 (*** *p* < 0.001) (Figure 3a). Concurrently, HU led to a biphasic response in HR. An important bradycardia occurred early (HU1, *** *p <* 0.001) reaching a maximum 11% decrease compared to C-1. This bradycardia persists to HU2 (*** *p <* 0.001) and then returned to baseline values on HU3, while conversely, a significant tachycardia was observed at recovery (*** *p <* 0.001) (Figure 3b).

HU induced an 85% reduction in mean locomotor activity, occurring immediately (HU1, *** *p <* 0.001) compared to C-1. This reduction remained throughout the entire HU until the end (HU5, *** *p* < 0.001). At recovery, locomotor activity returned to control values (Figure 3c). Mean subcutaneous temperature was significantly decreased during HU, from HU1 (*** *p <* 0.001) to HU5 (** *p <* 0.01), compared to C-1 and then returned to control values at recovery (Figure 3d).

Our results showed that HU induced inactivity, reduced food and water intake, a decrease in body weight, and a drop in subcutaneous temperature. The HR decreased in the first 2 days while the MBP increased moderately but significantly in the first 4 days. Reloading induced resting tachycardia and a decrease in MBP as a result of cardiovascular deconditioning.

### 3.2. ANS Activity Analysis

HRV was used to assess autonomic modulation of cardiac activity in both the temporal and spectral domains.

**Figure 4:**
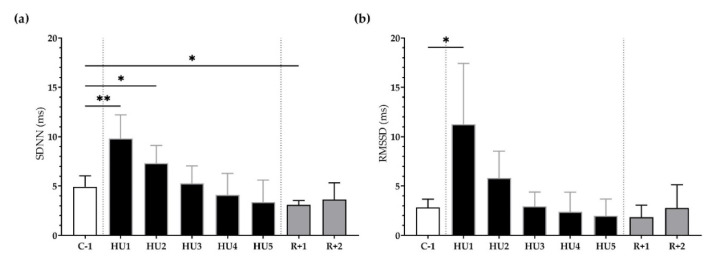
Time-domain analysis before (C-1), during unloading (HU1 to HU5) and recovery (R+1 and R+2). (**a**) Standard Deviation of NN intervals (SDNN) and (**b**) Root Mean Square of Successive R-R Interval Difference (RMSSD) were determined for 3 min mean triplicates, 20 h after each condition, between 8–10 am the next day. Inset: control (C-1, white bar), 5 days of unloading (HU1 to HU5, black bar) and 2-days recovery (R+1 and R+2, grey bar). Values are expressed as mean ± SD (*n* = 7 mice). * *p <* 0.05 and ** *p <* 0.01 vs. C-1.

First, in the time domain, the SDNN significantly increased following unloading at HU1 (** *p <* 0.01) and HU2 (* *p* < 0.05) as compared to the C-1. At recovery, SDNN significantly decreased at R+1 (* *p* < 0.05) (Figure 4a). Likewise, RMSSD was significantly increased at HU1 (* *p* < 0.05), compared to C-1 (Figure 4b).

**Figure 5:**
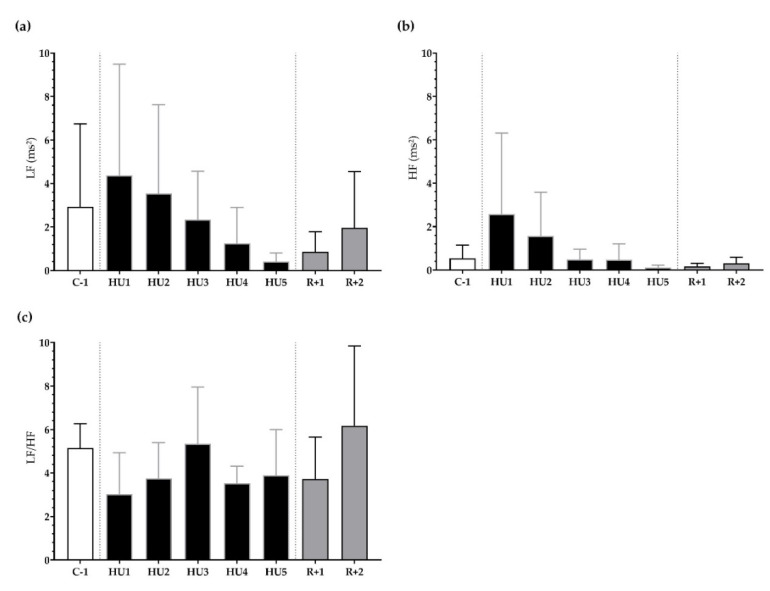
Frequency-domain analysis before (C-1), during unloading (HU1 to HU5) and recovery (R+1 and R+2). (**a**) low frequency (LF), (**b**) high frequency (HF), and (**c**) sympathovagal balance (LF/HF ratio) were determined for 3 min mean triplicates, 20 h after each condition, between 8–10 a.m. the next day. Inset: control (C-1, white bar), 5 days of unloading (HU1 to HU5, black bar) and 2-days recovery (R+1 and R+2, grey bar). Values are expressed as mean ± SD (*n* = 7 mice).

Next, frequency-domain analysis was performed. We found that LF, HF, and LF/HF were not significantly different during HU compared to control despite a trend for a decrease at HU4 of the unloading compared to C-1 for LF/HF (*p =* 0.08) (Figure 5a–c).

**Figure 6:**
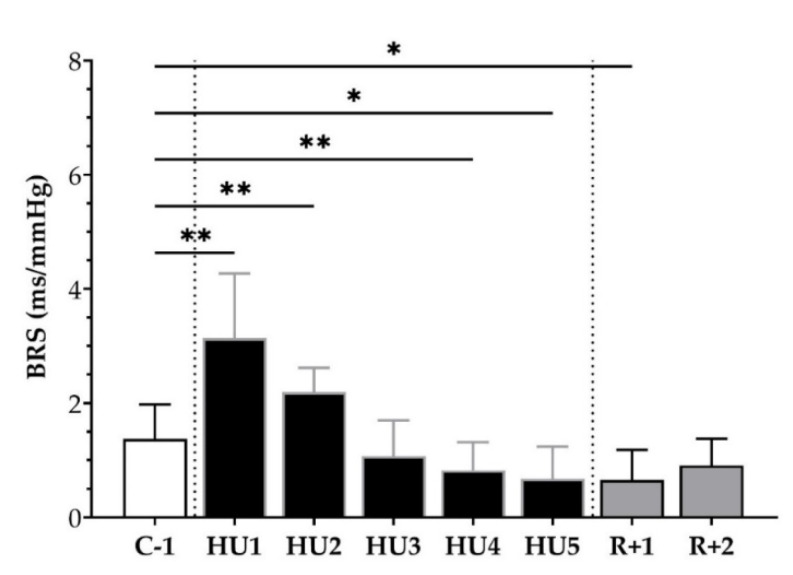
Baroreflex sensitivity (BRS) before (C-1), during unloading (HU1 to HU5) and recovery (R+1 and R+2). Inset: control (C-1, white bar), 5 days of unloading (HU1 to HU5, black bar) and 2-days recovery (R+1 and R+2, grey bar). Values are expressed as mean ± SD for *n* = 7 mice. * *p <* 0.05 and ** *p <* 0.01 vs. C-1.

We subsequently analyzed the BRS, using the sequence method, throughout the experimental protocol. An increase at HU1 (** *p <* 0.01) and HU2 (** *p <* 0.01) was observed with an ensuing decrease by HU4 (* *p* < 0.05) and HU5 (* *p* < 0.05), as compared to C-1. At the recovery, a decrease was observed at R+1 (* *p* < 0.05) compared to C-1 (Figure 6).

Altogether, HU induced an early increase in vagal activity and BRS. These parameters decreased during Reloading.

### 3.3. Phenotyping Circadian Rhythms

We next sought to further characterize the adaptations for each parameter measured by radio-telemetry over the entire experimental protocol.

#### 3.3.1. Locomotor Activity

**Figure 7:**
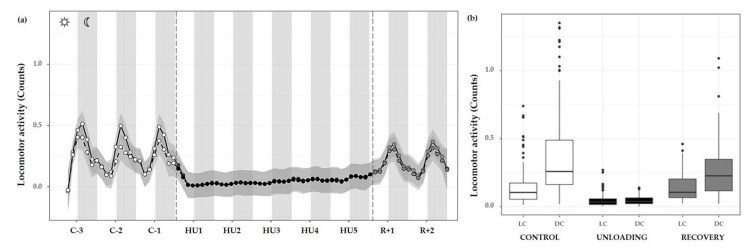
Locomotor activity changes during the unloading and recovery period. (**a**) Mean (black full curve) and median (black dotted curve) variations of locomotor activity were represented during 3 days of control (C-3 to C-1, white circle), 5 days of unloading (HU1 to HU5, black circle) and 2 days of recovery (R+1 and R+2, grey circle). White and grey bars indicate 12/12-h light (LC) and dark (DC) periods (7-7), respectively. Curves are smoothed, grey area represents the confidence interval (*n* = 8 mice). (**b**) Box plot showing locomotor activity during control (white box), unloading (black box) and recovery (grey box) periods for LC and DC (*n* = 8 mice).

Overall, the progression of locomotor activity during the control period showed rhythmic day/night cycles with maximum peaks during DC and minimum values in the LC. During HU, this rhythmicity was completely abolished and yet immediately retrieved during the recovery period (Figure 7a).

Locomotor activity was also assessed by 12 h means, and showed a marked activity pattern at expected nocturnal periods during the control condition (cycle effect, *** *p <* 0.001). These movements were significantly higher during DC than LC (** *p <* 0.01). As described, HU resulted in a striking decrease in locomotor activity, immediately following unloading and continued throughout HU (condition effect, *** *p <* 0.001). Locomotor circadian rhythm showed a significant difference between DC (** *p <* 0.01) and LC (** *p <* 0.01) compared to control DC and LC, respectively (interaction effect, *** *p <* 0.001). At the recovery, values similar to control locomotor activity and DC/LC oscillation were observed, with an increase in LC (*** *p <* 0.001) and in DC (*** *p <* 0.001) as compared to unloading (Figure 7b).

#### 3.3.2. Temperature

**Figure 8:**
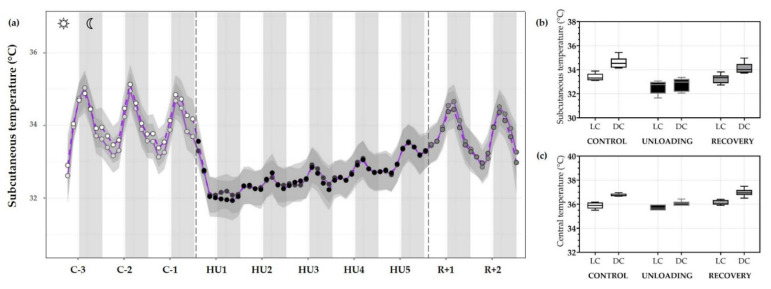
Subcutaneous and central temperature variations. (**a**) Mean (purple full curve) and median (purple dotted curve) variations of subcutaneous temperature were represented during 3 days of control (C-3 to C-1, white circle), 5 days of unloading (HU1 to HU5, black circle) and 2 days of recovery (R+1 and R+2, grey circle). White and grey bars indicate 12/12-h light (LC) and dark (DC) periods (7-7), respectively. Curves are smoothed, grey area represents the confidence interval (*n* = 8 mice). (**b**) Box plots showing subcutaneous (*n* = 8 mice) and (**c**) central (*n* = 6 mice) temperature variations for control (white box), unloading (black box) and recovery (grey box) for LC and DC.

Next, the subcutaneous temperature was plotted to visualize the evolution of temperature throughout the experiment. The temperature response to HU was similar to that observed for locomotor activity, with a consistent decrease in mean subcutaneous temperature, returning to normal upon recovery. During the control period, temperature showed rhythmic day/night cycles with maximum peaks during DC, and this rhythmicity was abolished during HU (Figure 8a).

Moreover, when analyses were performed by LC/DC means LC temperature during the control condition was lower as compared to DC temperature (*** *p <* 0.001) showing a circadian cycle effect (*** *p <* 0.001). In fact, unloading led to a decrease in DC temperature compared to DC during the control period (*** *p <* 0.001). As mentioned, mean subcutaneous temperature and cycle difference were decreased during unloading (condition effect, *** *p* < 0.001), inducing an interaction effect (*** *p* < 0.001). At the recovery, LC (*** *p* < 0.001) and DC (*** *p* < 0.001) differences were also restored compared to unloading and showed no difference compared to the control period (Figure 8b).

A similar trend for central temperature was also observed with a significant decrease during 5-days HU (condition effect, *** *p* < 0.001). Our results showed a significant decrease in DC temperature during unloading compared to the control period (*** *p* < 0.001) but no changes in LC. During the control period, LC (*** *p* < 0.001) and DC showed a circadian pattern (cycle effect, *** *p* < 0.001). This pattern is preserved, but attenuated, during HU (* *p* < 0.05). Core temperature returned to control values during recovery, showing no differences with the control period and a similar DC and LC pattern (*** *p* < 0.001) (Figure 8c).

#### 3.3.3. Arterial Blood Pressure

**Figure 9:**
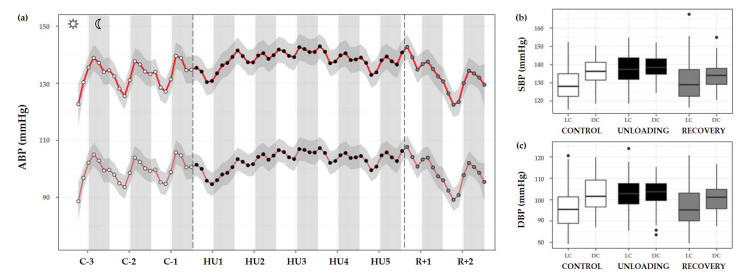
Arterial blood pressure changes before, during HU and recovery. (**a**) Mean variations of systolic (SBP, red full curve) and diastolic (DBP, red dotted curve) blood pressure were represented during 3 days of control (C-3 to C-1, white circle), 5 days of unloading (HU1 to HU5, black circle) and 2 days of recovery (R+1 and R+2, grey circle). White and grey bars indicate 12/12-h light (LC) and dark (DC) periods (7-7), respectively. Curves are smoothed, grey area represents the confidence interval (*n* = 8 mice). (**b**) SBP and (**c**) DBP box plots for control (white box), unloading (black box) and recovery (grey box) periods LC and DC.

ABP was separated into systolic and diastolic blood pressure (SBP and DBP, respectively). During the control condition, the circadian BP pattern is characterized by a decrease in both SBP and DBP during the day that reaches its trough around midday, with a subsequent gradual rise towards the nocturnal period with a peak near midnight. A continual circadian rhythm during the 3-day control period, with higher values during DC are observed, and both showed a cycle effect (*** *p* < 0.001). HU resulted in a significant increase in SBP (condition effect, *** *p* < 0.001) and DBP (* *p* < 0.05). There was also a marked loss of LC and DC rhythmic differences, SBP and DBP showed a significant interaction effect (*** *p* < 0.001) (Figure 9a–c).

SBP showed a significant difference in LC compared to DC in the control period (*** *p* < 0.001). During HU, LC and DC pattern was preserved (*** *p* < 0.001) by maintaining higher values in DC mean than LC mean. However, both LC and DC patterns during unloading showed significant differences compared to control period (*** *p* < 0.001). No differences were reported for DC in the recovery period compared to control despite a significant difference in LC (*** *p* < 0.001) (Figure 9b).

A similar pattern was observed for DBP, with a marked difference between LC and DC during the control period (*** *p* < 0.001). This pattern was maintained during HU (** *p* < 0.01). During unloading, LC was increased compared to control (*** *p* < 0.001) but no differences are reported concerning DC. At the recovery, LC and DC values were no different compared to the LC control period. There was a significant difference in LC (*** *p* < 0.001) and DC values (** *p* < 0.01) compared to unloading (Figure 9c).

#### 3.3.4. Heart Rate

**Figure 10:**
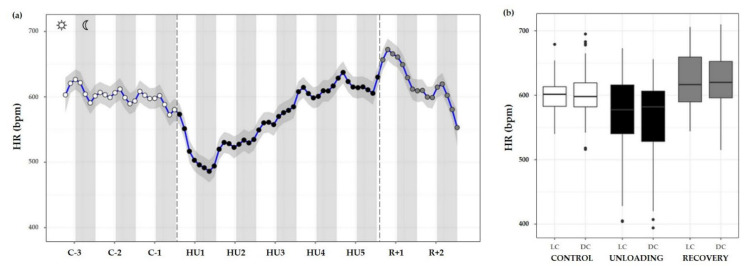
Heart rate responses before, during HU and recovery. (**a**) Heart rate (HR, blue curve) represented 3 days of control (C-3 to C-1, white circle), 5 days of unloading (HU1 to HU5, black circle) and 2 days of recovery (R+1 and R+2, grey circle). White and grey bars indicate 12/12-h light (LC) and dark (DC) periods (7-7), respectively. Curves are smoothed, grey area represents confidence interval (*n* = 8 mice). (**b**) Box plot of HR for control (white box), unloading (black box) and recovery (grey box) periods LC and DC.

HR measurements were also plotted as time series plots with a smoothing effect to better observe the patterns. Throughout the control condition, HR did not appear to follow a circadian pattern such as ABP (no cycle effect, ns). Unloading led to a significant decrease compared to the control (condition effect, *** *p* < 0.001). LC values during unloading were lesser than LC values during control (*** *p* < 0.001) or recovery period (*** *p* < 0.001). DC values followed the same trend, with a decreased value compared to control (*** *p* < 0.001) and recovery state (*** *p* < 0.001). LC and DC values during recovery were higher than control LC (*** *p* < 0.001) and DC (* *p* < 0.05) values (Figure 10a,b).

#### 3.3.5. Cosinor Analyses

Using the Cosinor analysis, the mesor, a rhythm-adjusted mean; the amplitude, the extent of predictable change within a cycle; and, the acrophase, a measure which can be defined as the time of day where the circadian cycle obtains its maximum, with respect to a fixed moment in time [31] were defined and presented in Table 1.

Data of locomotor activity and subcutaneous temperature are significantly changed during HU, showing a decrease in both amplitude and mesor value. During HU, our results showed a decrease in the amplitude of SBP and DBP, but also an increase in SBP (mesor) during HU. During the recovery period, data showed a decrease in SBP amplitude. HR showed a decrease in mesor value during HU and an increase during recovery.

For coherence in applying the model we further performed the rhythm detection test *p*-value, to determine the fit of the model to the data. It is noteworthy that the rhythm detection test showed that only locomotor activity and temperature fit the model during the control period. During the recovery period, the data fit the model only for temperature. Concerning BP the model was not able to fit the data.

Globally, HU led to a disrupted day/night rhythmicity of locomotor activity, temperature, and BP.

## 4. Discussion

### 4.1. Dynamic Responses of the ANS to HU and Recovery

In an effort to resolve some of the controversy surrounding how HU alters cardiovascular ANS function, we performed continuous recordings of the ABP, HR, locomotor activity, and temperature in a murine model of simulated microgravity. We found that the cardiovascular system responses were highly dynamic and that within the 5-days HU experimental phase, there was an initial adaptation followed by a relative return to baseline levels for HR and ABP. We observed that the initial response to HU consisted of a significant bradycardia, along with an increase in vagal activity and in BRS. Additionally, SDNN, a time-domain marker for HRV that reflects total ANS variability [34], showed a marked increase in HU1 and remained elevated up to HU2 as compared to control values. Correspondingly, the HRV time-domain marker, RMSSD, which is a marker of cardiac parasympathetic activity [34], was elevated on HU1. Even though these markers showed changes, it is of interest that no significant change was found in the frequency-domain analysis of LF, considered as an index of cardiac sympathetic and parasympathetic tones and the HF, reflecting only cardiac parasympathetic tone. HF tends to increase only on HU1 and couples with a decrease in HR, which indicate an increase in vagal tone. HRV in the frequency domain not reaching significance may be due to the large intra-individual variability [34], or even in the restrictive data time frame measured. The decrease in HR can hardly be explained by the reduction in locomotor activity, as reduced locomotor activity persists throughout the HU period. This change was accompanied by a decrease in water intake. This may reflect an early adaptation to the fluid redistribution to the upper part of the body mediated by neuro-hormonal mechanisms [35,36]. The increased central blood volume perceived by aortic and carotid baroreceptors and cardiac receptors induce an increase in natriuresis and diuresis leading to real hypovolemia [36]. An increase in plasma atrial natriuretic factor has been shown after 2 h of −30° HU in rats in response to early central hypovolemia [35]. Direct measurements of the central venous pressure in rats showed that an increase was related to the head-down level and that central venous pressure remained elevated after 24 h at −45° [37]. In our study, we suppose that the changes observed in HR as the initial response persisted mainly for the first 2–3 days, and then the animals recovered their baseline values are a reflection of the adaptation to central fluid shift [36]. Inverse to HR, ABP increased during this period, and also stabilized to baseline values by HU3. This included a stabilization of HR and HRV markers of vagal activity and also ABP values. A previous study also showed a moderate bradycardia by HU2 that continued over the course of 4 days, after which HR returned to baseline as measured by radiotelemetry in mice [13]. An increase in BP was also reported in rats by Tsvirkun et al. [22]. However, these authors did not report an initial decrease in HR. Several reasons may explain these discrepancies: for instance, the magnitude and the response to the cephalad fluid shift may vary with the species. Other studies have been performed at discrete timepoints, often when the animal is released from constraint, and therefore cardiovascular deconditioning is studied, or is confounded by the use of anesthesia for handling and experimentation [21,22,38,39,40].

The recovery period, which represents the return to normal gravity with the HU model, is also of interest to understanding post-flight adaptations in astronauts. Our data showed that, as expected, the recovery period provoked a significant resting tachycardia and a decrease in vagal activity. Indeed resting tachycardia in rodent models was previously described at recovery after 7 or 14 days HU in mice [13,41] and in rats [38,42,43,44]. These findings are in accordance with the central hypovolemia induced by the reloading and also leading to the increase in water intake significant on R+1. ABP levels returned to baseline values at R+1, but a significant decrease was observed at R+2. Our findings are in accordance with previous studies for the return to baseline by R+1. For example, resting ABP was normal following 14 days of HU in rats, but reached lower levels compared to the control group [43]. In mice, Powers and Bernstein found that ABP dropped slightly after reloading showing how increasing the timing of experimentation can have a large role in the results [13]. Indeed, most deconditioning studies are performed immediately following reloading of the animal, and several of these have shown ABP levels return to resting state. These data surrounding ABP responses to recovery, however, may differ and are likely due to the unloading duration, ABP measurement technique, and the duration of the recovery period following HU. The significant decrease in ABP at R+2 may reflect a reduced sympathetic response to postural challenge. It has been suggested that impaired baroreflex and sympathetic activity could play a role in cardiovascular deconditioning and orthostatic intolerance observed after exposure to simulated or real microgravity. Foley et al. [43] showed that baroreflex-mediated activation of sympathetic activity (measured by RSNA) was blunted by HU (13–15 days) in rats. In our experiment, we also observed that global variability (SDNN) and BRS were lower at the recovery than control period. However cardiac parasympathetic activity (RMSSD) showed no significant change. We did not find any modification in frequency-domain indicators of HRV and sympatho-vagal scale (LF/HF), which agrees with a previous study that showed simulated microgravity did not change autonomic balance assessed by spectral properties of BP and HR in rats [38,45]. However, other studies using pharmacological tools showed an alteration of the baroreflex mediated sympathetic activity and an altered HR sympathovagal balance (both a significant increase in sympathetic tone and a reduction in parasympathetic tone) in response to HU [34,39]. The decreasing trend observed in BRS may reflect a lack of ANS adaptation to postural challenges.

### 4.2. Light/Dark Cycles and Circadian Rhythms during HU and Recovery

Daily, 24 h rhythmic variations in body temperature and activity levels are found in mice with an increase in both during the DC [46,47]. Consistently with previous studies [48], we observed a positive correlation between temperature and locomotor activity. When measures of activity are elevated, there is also a rise in the temperature. Both of these parameters show a clear baseline day/night response with elevation during the DC versus the LC. This pair of parameters also correlate in terms of their response to the HU phase and their ability to re-acquire baseline waveforms during the recovery phase. Immediately upon introduction to the HU phase, mouse activity declined severely and lost any semblance of LC/DC periodicity; albeit to a greater degree during the DC when the mice are normally more active [47], and this is likely due to the restraint since these parameters returned to normal upon release. Temperature readings trended in a very similar pattern throughout the 5-day HU phase. Additionally, both of these parameters were able to regain LC/DC periodicity immediately upon exiting HU. Interestingly, mouse food uptake returned to baseline within 72 h during HU, suggesting a compensatory thermoregulation mechanism [49] possibly in order to maintain body temperature. Our finding for reduced body temperature is in contrast to a recent study conducted on astronauts that found an increase in core body temperature during spaceflights [50]. It is worth noting that the changes in intraperitoneal temperature and the subcutaneous temperature had the same pattern.

Furthermore, ABP and HR have an established circadian pattern with a peak during the DC in rodents [51]. In our results, we clearly see the circadian rhythm of BP during the control period, the perturbation of that rhythm during the days of HU, and the conduct of that rhythm during the days of recovery. The main finding is the most significant increase occurring in ABP during LC. This corresponds to a non-dipper pattern which may due to vascular sympathetic activation. This pattern has been already described in HU in rats by Tsvirkun et al. [22] who also reported the same pattern in isolated rats in individual cages. In addition, the possible changes in baroreflex and ANS regulation induced by the HU may also play a role. Indeed studies performed in sinoaortic denervated rats have shown that the disruption of the baroreflex elevates the BP in rats during the light period, eliminating the 24-h rhythmicity although the level and pattern of locomotor activity rhythms were not changed by sinoaortic denervation [52].

For example, ABP shows a circadian waveform during the control phase, and the changes observed during HU are subtler and more difficult to interpret. There appears to be an alternation in the shape of the waveform immediately upon introduction to HU that then leads to a distortion in the circadian waveform on each subsequent day. Even upon exit to the recovery phase, it was not evident during the 2 days of recovery how the ABP waveform would eventually regain the baseline shape. In fact, the circadian rhythm observed for the days R+1 and R+2 are more different than any subtle modulations observed during the 3 control days. The circadian cycle of HR, after a complete perturbation on HU1, appears to gradually increase over the remaining days of HU. Akin to the recovery response observed for ABP, HR was not able to return to a baseline waveform within the recovery period.

Finally, we found that locomotor activity and temperature that fits the model during the control period no longer fit the model during HU, which was probably related to disruptions in circadian rhythms. However, fitting the model to certain data such as BP was not possible even during the control period. We suppose that these results may be influenced by the number of days of control versus the HU and the recovery phase. Second, we presume that since the period was tightly controlled in our experiment, this may bias the wave function. Future work and more thorough analyses are required to fully understand how HU impacts on the circadian cycle.

### 4.3. Limitations of the Model and Analyses

While we conducted a controlled experiment to understand the impact of HU on cardiovascular conditioning, we found considerable interaction between the physiological parameters, food and water consumption, with HU. Stress responses are classically measured by the loss of body weight, food consumption, and decreased core body temperature; all of which were altered in our experiment. It is important to note that mice were housed individually in HU studies. Social isolation and HU are known to induce stress, anxiety, depressive-like behavior, as well as disrupted circadian rhythms [22,38,53]. Although we tried to reduce stress, by acclimating the mice to handling and isolation prior to experimentation, we cannot exclude the effects of stress on the cardiovascular responses observed. There is some consensus that stress-related features manifest in unloading models and these can influence ABP and HR levels [22,54]. As such, the ensemble of changes renders the dissection of the root cause of cardiovascular ANS alterations during HU, and the ensuing deconditioning, challenging. These shortcomings are known in the literature and remain a caveat for all rodent-based studies and may also contribute to the disputed nature of findings.

Despite these limitations, our findings offer insight into the fluxes in HR and ABP in response to the experimental phase and the time that may explain the conflicting findings in the field. One noteworthy observation in our study is that several parameters of ANS activity appear to return to control period levels during the HU period. While we are aware that the exposure to microgravity in our experiment was of a relatively short duration, our findings agree with previously published studies of short-term HU showing similar adaptations [13,22]. It is important to keep in mind that most of the experimental protocols used assess cardiovascular ANS tone at discrete time points, and several of these require the unloading of the animal and/or the use of anesthesia. Moreover, analyses of the different parameters of the ANS require stable HR and ABP signals, which are a challenge to obtain in awake, free-roaming animals. Thus, our analyses of the ANS indices followed those previously published for 3 min intervals in the early morning [28]. In addition, our data equally shows a divide between locomotor activity counts and the cardiovascular markers, HR and ABP. These findings are perplexing given the high correlation between HR and activity (reviewed but not limited to [55,56]). We hypothesize that the observed overall increase in the sympathetic tone during the LC may help explain these findings. We need to keep in mind that in addition to the ground-based nature of these experiments, there are other features such as the fact that mice are quadrupeds and that fluid redistribution towards the upper part of the body may not be as significant as in Humans [13]. Although the murine model is a suitable model for assessing cardiovascular adaptation to microgravity such as observed in bed rest and spaceflight studies, the interpretation of the data should be made cautiously between species. It is noteworthy to note that 1G gravity applies to this model unlike spaceflight [57].

### 4.4. Conclusions and Potential New Insights

In our study, we demonstrated the several phases of physical, cardiovascular, and autonomic adaptation to simulated microgravity. On the one hand, we demonstrated an initial bradycardia associated with an increase in cardiac vagal activity and BRS, accompanied by a decrease in water intake, which may reflect an early adaptation to fluid redistribution. HU also led to a disruption of cardiovascular circadian rhythms, in particular a decrease in day/night differences in BP characterized by an increase in BP in LC, which may be related to an increased sympathetic activity. On the other hand, our results confirm that HU induced an immediate, dramatic, and persistent decrease in locomotor activity. Surprisingly, HU also induces a decrease in subcutaneous and core temperature, which to our knowledge has not been shown in mice. Finally, at the recovery, the mouse showed a resting tachycardia with a decrease in BP, in cardiac vagal activity, and BRS, reflecting the re-adaptation to normal gravity. Our investigation with continuous monitoring has both provided a degree of clarity with regard to the cardiovascular adaptation and regulation by the ANS in HU, also confirm the interest of this model to simulate and assess the post-flight cardiovascular deconditioning.

While the circadian rhythm is the most studied cycle, there are also cycles in nature that are shorter, ultradian (<22 h), and longer, infradian (>26 h) that also play a role in the regulation of biological systems [58]. The underlying mechanisms for infradian rhythms are poorly understood even if examples of these, which include levels in sex hormones [59,60], melatonin [61], and dopamine [62] have been shown. A major issue in the study of such cycles is the enormous amount of interaction between all the different rhythms with all their various periods. For instance, dopamine, an important neurotransmitter involved in stress and depression [63], not only exhibits ultradian rhythms but also influences circadian and infradian rhythms [62]. Dopamine levels were found to be reduced in mice exposed to 1 month of spaceflight on the Russian biosatellite BION-M1 [64], suggesting possible interactions between a known ultradian rhythm and microgravity. Studies have implicated both ultradian and infradian rhythms play a role in HRV [65]. Integrating our database with existing databases, coupled with new findings, and tracking in real-time physiological and behavioral parameters are key areas of study in the future to understand and enhance cosmonaut physiology for spaceflight missions.

## Figures and Tables

**Table 1:** Cosinor analysis of mesor, acrophase, and amplitude, before, during and after unloading.

	Condition		
	Control	Unloading	Recovery
**Locomotor activity (Counts)**			
*MESOR*	0.26 ± 0.11	0.04 ± 0.01 ***	0.21 ± 0.05 ###
*Acrophase*	−1.10 ± 0.82	−0.64 ± 0.68	0.95 ± 1.00 *** ##
*Amplitude*	0.18 ± 0.11	0.01 ± 0.01 ***	0.13 ± 0.08 ##
*Rhythm detection test p-value*	0.005 *	*0.420*	*0.070*
**Subcutaneous temperature (°C)**			
*MESOR*	33.81 ± 0.72	32.69 ± 0.51 ***	33.73 ± 0.40 ##
*Acrophase*	−0.69 ± 0.25	−0.72 ± 1.01	0.18 ± 1.36
*Amplitude*	0.78 ± 0.19	0.23 ± 0.07 ***	0.71 ± 0.17 ###
*Rhythm detection test p-value*	0.004 *	*0.129*	0.026 *
**Systolic blood pressure (mmHg)**			
*MESOR*	128.3 ± 14.4	134.4 ± 14.9 *	129.0 ± 12.8
*Acrophase*	−0.76 ± 0.96	−0.41 ± 0.39	−0.26 ± 1.06
*Amplitude*	6.32 ± 2.74	2.66 ± 1.28 ***	3.39 ± 1.89 **
*Rhythm detection test p-value*	*0.118*	*0.192*	*0.486*
**Diastolic blood pressure (mmHg)**			
*MESOR*	102.3 ± 2.8	106.4 ± 7.1	101.8 ± 8.6 #
*Acrophase*	−1.18 ± 0.14	−0.40 ± 0.64	−0.05 ± 1.25 *
*Amplitude*	5.37 ± 2.53	2.09 ± 1.10 **	2.28 ± 4.95
*Rhythm detection test p-value*	*0.104*	*0.316*	*0.372*
**Heart rate (bpm)**			
*MESOR*	590.6 ± 14.5	569.9 ± 22.9 *	616.6 ± 22.2 * ###
*Acrophase*	0.30 ± 0.87	0.17 ± 0.97	−0.09 ± 0.66
*Amplitude*	18.23 ± 8.29	8.57 ± 6.01	16.47 ± 10.46
*Rhythm detection test p-value*	*0.170*	*0.562*	*0.486*

Cosinor analysis was performed for locomotor activity (counts), subcutaneous temperature (°C), systolic blood pressure (mmHg), diastolic blood pressure (mmHg), and heart rate (bpm). Mean of mesor, acrophase, amplitude and model coherence, were determined for the control period of 3 days, hindlimb unloading period of 5 days and the 2 days of recovery. Rhythm detection test refers to the fit of the model to the data where *p* < 0.05. Data were expressed as mean ± SD (*n* = 8). * *p* < 0.05, ** *p* < 0.01 and *** *p* < 0.001 vs. Control; # *p* < 0.05, ## *p* < 0.01 and ### *p* < 0.001 vs. Unloading.

## Data Availability

All data related to the article are found within the article and the 2 h time bin datasets are available at doi:10.57745/QVRW8W (7 February 2022).
